# High-Dose Radioiodine Therapy Did Not Result in Better Thyroglobulin Decline in Patients with Extra-Thyroid Tumor Extension of Papillary Thyroid Cancer

**DOI:** 10.1055/s-0045-1802954

**Published:** 2025-03-26

**Authors:** Mohamad Ghazanfari Hashemi, Mohsen Bakhshi Kashi, Mohammad Reza Ghasri, Saeed Farzanefar, Yalda Salehi, Mehrshad Abbasi

**Affiliations:** 1 Department of Nuclear Medicine, Imam Khomeini Hospital Complex, Tehran University of Medical Sciences, Tehran, Iran; 2 Department of Radiology, Cancer Institute, Tehran University of Medical Sciences, Tehran, Iran; 3 Research Center for Nuclear Medicine, Tehran University of Medical Science, Tehran, Iran

**Keywords:** extra-thyroid extension, PTC, radioiodine, therapeutic, thyroglobulin

## Abstract

**Objective:**

In this study, the response to treatment in patients with extra-thyroid extension (ETE) of papillary thyroid cancer (PTC) was compared between different radioiodine treatment doses.

**Methods and Materials:**

In this retrospective cross-sectional study, patients with pathology-proven ETE who were hospitalized for radioiodine therapy from December 2015 to May 2018 at a referral university hospital were identified. Demographic data, radioiodine doses, and off-levothyroxine thyroglobulin and antithyroglobulin levels, before and after treatment, were collected. Alterations in thyroglobulin levels before and after treatment were compared between patients receiving different doses of radioiodine.

**Results:**

Sixty patients were analyzed (mean age: 44.1 ± 14.4 years; 61.7% females). On average, the thyroglobulin levels were 59.1 ± 92.0 and 45.7 ± 81.5 ng/mL at baseline and after treatment, respectively. The thyroglobulin levels decreased from 6.2 ± 6.3 to 1.7 ± 0.2 ng/mL ( *p* = 0.510), 55.8 ± 101.3 to 11.5 ± 17.2 ng/mL ( *p* = 0.07), and 62.8 ± 91 to 60.9 ± 93.1 ng/mL ( *p* = 0.83) in the 100- to 149-, 150- to 199-, and 200- to 250-mCi iodine therapy groups, respectively. Treatment with doses of less than 200 mCi were significantly more effective in reducing posttreatment thyroglobulin levels compared with higher doses ( *p* = 0.05). In the subgroup analysis, nonmetastatic cases treated with less than 200 mCi iodine had significantly greater thyroglobulin reduction compared with metastatic patients treated with ≥200 mCi iodine ( *p* = 0.05). Macroscopic (vs. microscopic) invasion into adjacent tissues had no impact on thyroglobulin decrease.

**Conclusion:**

The administration of higher radioiodine doses for the treatment of PTC patients with ETE does not yield additional therapeutic benefits in terms of posttreatment thyroglobulin reduction.

## Introduction

Papillary thyroid cancer (PTC) is the most frequent endocrine malignancy. 1 2 This cancer generally presents with an indolent nature and favorable prognosis. 3 However, a small portion of patients experience metastasis and local recurrence. 3 There are a few risk factors at presentation that predict aggressive tumors. Among these, the presence of extra-thyroid extension (ETE) in the pathology of thyroidectomy is a relatively rare but significant factor. ETE is defined as tumor invasion into extra-thyroid tissue, including muscles or cartilage. It is considered a notable risk of recurrence. 4 Even minimal ETE may decrease survival. 5 Metastasis mainly occurs in the lung and bone. 6 7 Interestingly, many patients with diffuse lung metastasis are considered curable with iodine therapy. 8 Local recurrence often involves lymph node metastasis, which does not change the overall prognosis of the patient and is essentially considered curable. 9 Local invasions into the trachea and larynx, esophagus, and the carotid sheet are serious events with rather poor prognosis. 9 Local recurrence and invasion of neck structures start from tumor penetration of the thyroid capsule and soft tissue invasion around the thyroid gland. 5 10 This ETE is considered a significant risk factor for future recurrence. 5

Radioiodine therapy provides potent targeted radiation therapy with minimal side effects. 11 12 There is controversy regarding the selection of the best dose for radioiodine therapy. In the absence of nodal or distance metastasis, 25 mCi is found to be the smallest effective dosage for remnant ablation 13 ; nonetheless, it is generally accepted that higher iodine doses should be administered for patients with higher risk of recurrence. As mentioned above, ETE predicts high risk of recurrence. In the current study, we assessed the outcome of radioiodine doses higher than the usual recommended doses. The outcomes of different therapeutic doses in the first radioiodine therapy session are compared. The subsequent thyroglobulin levels at follow-up were the main endpoint.

Thyroglobulin levels correlate well with the bulk of the remnant or recurrent tumors and can serve as a surrogate for recurrence or persistent disease. In brief, we aim to assess the efficacy of higher radioiodine doses during the first radioiodine therapy in patients with PTC and ETE. The first iodine therapy was focused on, because it is probably more important than subsequent radioiodine treatment sessions. 11

## Methods

In a retrospective cross-sectional design conducted at the Valiasr Hospital, a tertiary referral teaching university hospital, the treatment files of patients treated with radioiodine for PTC from December 2015 to May 2018 were reviewed. About 2,700 patients, including new cases and repeated treatments, were treated during this period. The center was one of Iran's five inpatient radioisotope wards at that time. The indication for radioiodine treatment and radioiodine dose selection was generally based on the American Thyroid Association (ATA) guidelines from 2009 and later 2015. Accordingly, patients at moderate and high risk of recurrence were treated with radioiodine. Papillary thyroid microcarcinomas (PTMCs) were not treated with radioiodine. Nevertheless, bifocal or multifocal PTMC with a total tumor size exceeding 1 cm was often treated contrary to the ATA recommendations. Patients receiving more than 30 mCi of radioiodine were hospitalized based on the local radiation protection protocols. Certain additional institutional treatment criteria were considered in addition to ATA recommendations as follows: lymph node involvement, lymphovascular invasion, capsular invasion, and neural invasion were treated with fixed doses of 150 mCi unless another factor (i.e., high creatinine, age >70 years, or body weights <50 kg) indicated otherwise. Patients with treatment-morning thyroglobulin levels higher than 50 ng/mL were also treated with doses of 150 to 200 mCi unless the previously mentioned limiting factors were presented. It is worth mentioning that as a routine protocol in our center, with regard to the ATA recommendations, proper low-iodine diet was administered for patients prior to radioiodine therapy aiming to enhance radiotracer uptake. 12 14 15

### Enrolment Procedure

The inclusion criteria for participant enrollment were as follows: (1) pathology-confirmed PTC, (2) presence of ETE in the pathology reports from surgeries performed before the first radioiodine therapy, and (3) availability of data at the 6-month follow-up. The exclusion criteria included patients younger than 20 years and those with suspected bulky lung, bone, or brain metastases based on stimulated thyroglobulin levels (>2,000 ng/mL) on the day of radioiodine therapy.

In the current study, ETE was defined as any type or amount of microscopic or macroscopic soft tissue invasion around the thyroid gland, including fat, muscle, nerve, viscera (e.g., trachea or esophagus), and cartilage. Patients with follicular thyroid cancer, medullary thyroid cancer, Hurthle's cell carcinoma, and poorly differentiated cancers were not included. In total, follow-up data for 67 patients with ETE were available. Three patients younger than 20 years were excluded. Four patients were also excluded due to outlier thyroglobulin levels (>2,000 ng/mL) and the final analysis was conducted on the data of 60 patients (37 females; 61.7%). In addition to demographic data and radioiodine doses, off-levothyroxine thyroglobulin and antithyroglobulin levels on the day of the first radioiodine treatment and at 6-month follow-up visit were collected. The indication for radioiodine therapy and the selected radioiodine dose varied among the attending physicians and during the study. Three nuclear physicians worked in the nuclear medicine ward during the study period. Two of them treated ETE with doses of 200 to 250 mCi and the other used iodine doses less than 200 mCi (i.e., 100–175 mCi). Table 1 provides an explanation of the factors influencing the decision regarding the administered dose of radioiodine.

**Table 1:** Participants' health and tumor characteristics

	Category	Frequency (%) or mean (SD)
Gender	Female	37 (61.7)
Age (y)		44.1 (14.4)
Stage	I	35 (58.3)
	II	16 (26.7)
	III	4 (6.7)
	IV	5 (8.3)
T stage	0–2 cm	26 (43.9)
	2–4 cm	16 (26.3)
	> 4 cm	18 (29.8)
Capsular invasion	Negative	29 (48.3)
	Indeterminate	2 (3.3)
	Positive	29 (48.3)
Lymph node invasion	Negative	15 (25.0)
	Indeterminate	2 (3.3)
	Positive	43 (71.7)
Lymphatic or vascular angioinvasion	Negative	25 (41.7)
	Indeterminate	4 (6.7)
	Positive	31 (51.7)
Margin involvement	Negative	51 (85)
	Positive	9 (15)
Extra-thyroid extension	Microscopic	52 (86.7)
	Macroscopic	8 (13.3)
Size (mm)		31.9 (21.1)

Note: Staging is provided based on the 2017 AJCC Cancer Staging Manual and employing pathology data for tumor size/lymph node invasion and imaging for metastasis status.

Data are frequency or mean and percentage or standard deviation (SD) in the parentheses.

Lung metastasis and/or bone metastasis were determined based on posttherapy whole-body iodine scans. Follow-up data were collected from the 6-month visit records. Changes in thyroglobulin were analyzed before and after treatment between patients treated with high- and low-dose iodine therapy. Given that dose selection was oriented around thyroglobulin levels and metastasis status, stratified analysis was conducted in patients with and without metastases separately. The research protocol was conducted in strict accordance with the ethical principles set forth in the Declaration of Helsinki. SPSS v26 was employed, with significance level set at less than 5%.

## Results

**Fig. 1:**
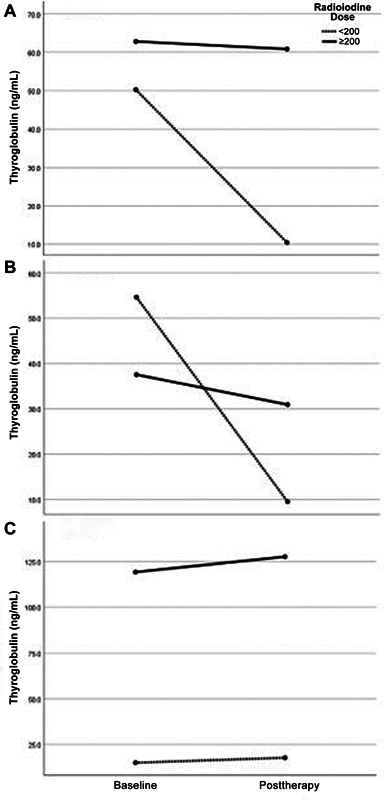
Thyroglobulin change with radioiodine therapy in different dose groups with respect to the presence of metastasis. ( **A** ) All patients. ( **B** ) Patients without metastasis. ( **C** ) Patients with metastasis. Extra-iodine doses had no additional benefit even in metastatic patients. Treatment with doses less than 200 mCi were more effective to reduce thyroglobulin levels compared with higher doses ( *p* = 0.05).

The health characteristics of patients and tumor specifications are presented in Table 1 . Thyroglobulin levels were 59.1 ± 92.0 and 45.7 ± 81.5 ng/mL at baseline and after treatment, respectively. The change in thyroglobulin levels in correlation with different therapeutic doses is presented in Table 2 . Changes in thyroglobulin by treatment are depicted in Fig. 1 . Patients who received radioiodine doses less than 200 mCi showed remarkable normalization of thyroglobulin levels after treatment compared with those treated with 200 mCi or higher doses ( *p* = 0.05; partial eta-squared = 0.06 and estimated power = 0.49). In patients without metastasis, including 16 patients who received less than 200 mCi and 29 who received ≥200 mCi, not only was the initial thyroglobulin level higher in the lower-dose group but the extent of the thyroglobulin reduction was also significantly greater in this group ( *p* = 0.05; partial eta-squared = 0.06 and estimated power = 0.49). In patients with metastasis ( *n* = 2, <200 mCi; and *n* = 13, ≥200 mCi), although the baseline thyroglobulin level was higher in those treated with a higher dose, treatment did not alter the posttherapy thyroglobulin change in either group.

**Table 2:** Thyroglobulin decline in different radioiodine treatment dose subgroups in regard to presence or absence of systemic metastasis

		Nonmetastatic			Metastatic			Total		
Thyroglobulin (ng/mL)		Baseline	Follow-up	Sig.	Baseline	Follow-up	Sig.	Baseline	Follow-up	Sig.
Radioiodine treatment dosage	100–149 (100 ± 0) *n* = 2	10.6 (0)	1.5 (0)	– a	1.7 (0)	1.9 (0)	– a	6.2 (6.3)	1.7 (0.2)	0.51
	150–199 (154 ± 10.1) *n* = 16	57.6 (104.6)	10.0 (16.7)	0.07	28.5 (0)	33.8 (0)	– a	55.8 (101.3)	11.5 (17.2)	0.07
	200–250 (217.9 ± 24.2) *n* = 42	37.6 (67.4)	30.9 (67.1)	0.42	119.2 (112.5)	127.6 (110.0)	0.73	62.8 (91.0)	60.9 (93.1)	0.83
	Total (197.1 ± 39.3) *n* = 60	43.6 (80.6)	23.3 (55.3)	0.05	105.3 (110.6)	113 (109.1)	0.71	59.1 (92.0)	45.7 (81.5)	0.144

Note: The significance level (i.e., sig.) was calculated employing paired *t* -test.

a Few sample.

Eight patients (13.3%) had macroscopic invasion of the skeletal muscle, trachea, esophagus, and recurrent laryngeal nerve. No remarkable interaction effect was detected between the presence of microscopic or macroscopic extra-thyroid invasion and thyroglobulin decrease.

## Discussion

We documented that higher doses of radioiodine in the patients with ETE did not result in a reduction of thyroglobulin levels at the 6-month follow-up. Patients with treatment doses of 150 to 199 mCi had the best thyroglobulin response to radioiodine therapy. There was no evidence of interaction effect on thyroglobulin decrement between patients with or without metastasis or those with macroscopic versus microscopic extra-thyroid invasion.

**Fig. 2:**
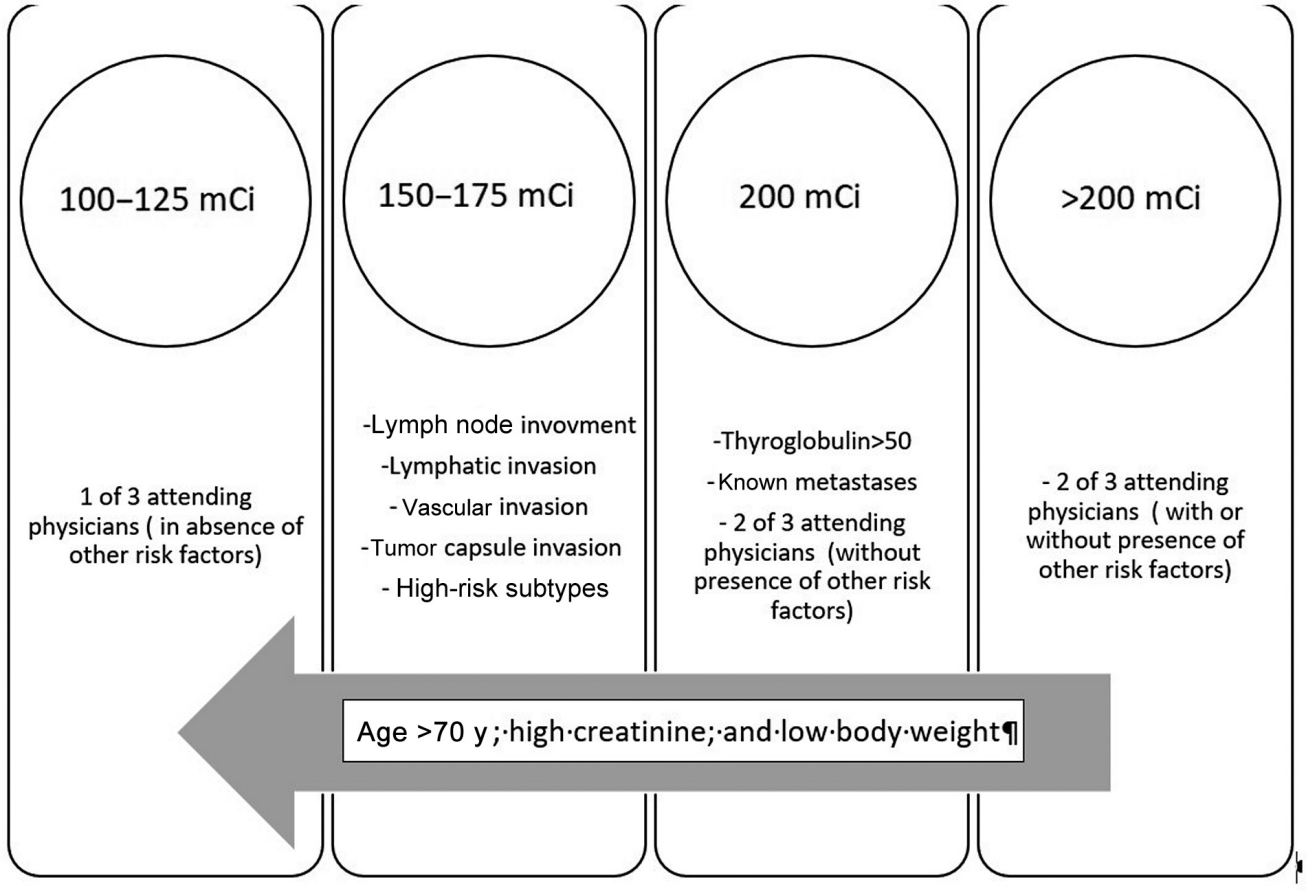
Radioiodine dose selection clues for study participants, patients with papillary thyroid cancer, and extra-thyroid tumor extension.

For decades, radioiodine doses have been determined empirically. 12 16 As a general concept, the first radiation dose plays a crucial role in eradicating the remnant tumor cell and debris to result in a better prognosis. 12 13 14 This could be attributable to post-first-course therapy radioresistance. 12 15 Changes in blood vessels after the first radiation course may reduce future blood-mediated medication supply to the tumor. Additionally, intracellular genetic changes and activation of cell protection mechanisms may cause radioresistance at the cellular level. 16 17 Furthermore, higher radioiodine doses have been administered for patients with ETE and for those with macroscopic ETE compared with microscopic ETE. 17 On the other hand, the ATA guidelines recommend that patients with microscopic ETE are at moderate risk of recurrence and could be treated with doses of 30 to 150 mCi, while higher doses are recommended for patients with macroscopic ETE at high risk of recurrence. Based on these considerations, we chose higher doses for the patients with extra-thyroid invasion. In this study, we enrolled patients with a poor prognosis, reflected in consequent less thyroglobulin reduction after therapy. Still, the effect of an additional higher dose on thyroglobulin reduction did not support the use of such an additional dose. As depicted in Fig. 2B , nonmetastatic patients who received less than 200 mCi radioiodine doses and had higher pretreatment thyroglobulin levels showed greater thyroglobulin reductions compared with those who received ≥200 mCi radioiodine doses. There are shreds of evidence that radioiodine can be escalated up to 600 mCi per dose with dosimetry support for the patients. 17 Nevertheless, no notable patient-oriented achievement has been documented for these higher doses to date. Table 3 presents three similar studies that employed different radioiodine doses in lower-risk patients than those in the current study, where no benefit was detected with higher administered radioiodine doses.

**Table 3:** Review of the literature concerning proper radioiodine dose in extra-thyroidal extension of papillary thyroid cancer

Study	*N*	Inclusion criteria	Comparison	Result
Han et al 19	176	• Tumor size ≤2 cm • Microscopic ETE • No cervical LN metastasis	30 vs. 150 mCi (retrospective)	No significant difference in success rates between the low-dose (59/61, 97%) and high-dose groups (22/24, 92%; *p* = 0.30)
Seo et al 20	180	• Tumor size ≤2 cm • Microscopic ETE • Positive central LN metastasis	30 vs. 80 mCi (retrospective)	No significant differences in the responses to ablation ( *p* = 0.810) and long-term outcomes ( *p* = 0.663)
Zhang et al 21	102	• Complete thyroidal resection, macroscopic ETE, any N stage, and stimulated thyroglobulin > 5 ng/mL when thyroglobulin antibodies were low	30 vs. 100 mCi (randomized)	No significant difference in the 6-mo recurrence or ablation rate

Abbreviations: ETE, extra-thyroid extension; LN, lymph node.

In summary, although ETE worsens outcome in terms of lymph node and distant metastasis and reduced survival, 4 5 the response rate to higher versus lower radioiodine doses appears similar in patients with ETE. 18 Consequently, ETE could be considered an unmodifiable risk factor for future recurrence.

Our study has certain limitations. The number of patients in the low-dose radioiodine groups was limited. Considering that ETE of PTC is rare, this inherited limitation is inevitable. The 60 cases analyzed here were drawn from 2,700 radioiodine treatments at the center over 3 years. Second, this study was retrospective. The dose selections were based on the clinical decisions of the treating physicians (i.e., Y.S., S.F., and M.A.) and not strictly on specific criteria. Consequently, unknown or uncollected variables are certainly present. We believe that these variables did not affect the study's main result, indicating no remarkable change in thyroglobulin levels in high-risk patients who received high radioiodine doses. To obtain more precise results, a randomized clinical trial should compare different radioiodine doses in patients with extra-thyroid tumor extension. We do not recommend such a study based on the result of the current study since we found no benefit for higher-dose prescriptions. Finally, it is not appropriate to draw definitive conclusions about the treatment response based solely on stimulated thyroglobulin levels without ultrasonography results, which we did not collect. Future studies may provide further insight by comparing the efficacy of radioiodine therapy for microscopic versus macroscopic ETE using both thyroglobulin levels and ultrasound findings.

## Conclusion

In conclusion, treating patients with extra-thyroid PTC extension with higher radioiodine doses in the first treatment course provides no benefit in terms of additional thyroglobulin reduction.
